# Research on the impact of multidimensional poverty in childhood on disability in middle-aged and older adult

**DOI:** 10.3389/fpubh.2025.1429457

**Published:** 2025-04-15

**Authors:** Ruirui Xie, Fei Zhao, Lili Ding, Huiling Dong, Bingyi Wu

**Affiliations:** ^1^Department of Public Health, Shandong Second Medical University, Weifang, China; ^2^School of Management, Shandong Second Medical University, Weifang, China; ^3^School of Nursing, Shandong Second Medical University, Weifang, China

**Keywords:** childhood, multidimensional poverty, middle-aged and older adult, disability, A-F double identification method of poverty, propensity score matching

## Abstract

**Objective:**

This study measured the incidence of multidimensional poverty in childhood and decomposed the poverty index to explore the impact of multidimensional poverty in childhood on disability in middle-aged and older adult.

**Methods:**

The A-F poverty double identification method was employed to assess children's multidimensional poverty, and in accordance with standard scientific research methods, its influence on the disability status of middle-aged and older adult was analyzed through Probit regression. Robustness testing was conducted using the propensity score matching method.

**Results:**

(1) Multidimensional poverty in childhood increased the probability of disability among middle-aged and older adult by about 4.2%. (2) Heterogeneous results indicated that childhood multidimensional poverty had differential urban-rural impacts on the disability status of middle-aged and older adult (*P* < 0.05). (3) Multidimensional poverty in childhood had long-term negative consequences for both men and women, with significant adherence (*P* < 0.05). In contrast, the negative impact on women's health status was greater.

**Conclusions:**

Children's needs vary across generations. In times of extreme resource scarcity, childhood poverty is primarily related to a lack of material resources. Multidimensional poverty in childhood affects the health status of middle-aged and older adult mainly through deprivation of material resources.

## Introduction

Childhood poverty remains a critical barrier to human capital formation, with 1.1 billion people experiencing multidimensional poverty globally. Over half are children, concentrated in sub-Saharan Africa (56% poverty rate) and South Asia (30%) (1). China's poverty alleviation experience provides unique insights for developing economies: its Targeted Poverty Alleviation Campaign (2015–2020) lifted 13 million children from poverty through systemic interventions (2), while its urban-rural dual structure (65.2% urbanization rate) offers a natural laboratory for studying intergenerational poverty transmission (3). These empirical observations necessitate a corresponding evolution in poverty assessment frameworks.

The understanding of poverty has shifted from income-based measures (4) to a multidimensional framework that encompassing wellbeing aspects such as life expectancy, freedom, and security (5, 6). In assessing childhood poverty, the traditional income-centric approach falls short of capturing crucial factors influencing child welfare (7). Scholars such as Gordon D and Cummins have proposed multidimensional indicators (8, 9), while Chinese researchers like Li and Yang have developed frameworks for understanding childhood poverty (10). Although the above studies provide a rich theoretical basis for understanding child poverty, empirical research in China is relatively scarce, and related work mainly relies on theoretical frameworks developed abroad. Studies indicate that childhood multidimensional poverty affects adult health (11), education, and income (12). However, research on the long-term health effects of childhood multidimensional poverty on middle-aged and older populations remains limited in China. To address this gap, this study adopts the life course theory to examine the impact of childhood multidimensional poverty on disability in later life stages. The theoretical framework posits that early-life adversities significantly shape health trajectories in adulthood, a perspective strongly supported by empirical evidence. This integration of theory and data provides a robust foundation for elucidating the mechanisms through which childhood deprivation influences lifelong health outcomes.

In this study, we used data from the China Health and Retirement Longitudinal Study (CHARLS) to investigate childhood multidimensional poverty. We assessed both its prevalence and severity, decomposing poverty dimensions. Our approach went beyond income-based metrics and incorporated a comprehensive multidimensional child poverty index system, aligning with established scientific practices. This system evaluated children's deprivation across dimensions like health, nutritional status, income, and social interactions during their formative years. Building on this framework, we thoroughly analyzed how childhood multidimensional poverty affected the health of middle-aged and older adult, aiming to provide relevant recommendations for addressing child poverty from a life-course perspective.

## Materials and methods

### Data sources

This study employed data from the China Health and Retirement Longitudinal Study (CHARLS) to examine the influence of multidimensional childhood poverty on the functional disability status of middle-aged and older adult. The multidimensional childhood poverty status was derived from the 2014 Life Course Survey data, while the functional disability status of the middle-aged and older adult was drawn from the 2018 dataset. The two years of survey data were merged and processed using the “tracking ID number” method.

The CHARLS data is a comprehensive and wide-ranging nationally representative longitudinal survey conducted and developed jointly by the National Development Research Institute of Peking University and the China Social Science Survey Center of Peking University. Its main focus is on collecting information from Chinese individuals aged 45 and above. The CHARLS 2014 Life Course Survey represents a retrospective investigation that provides a more comprehensive depiction of the life experiences and developmental trajectories of middle-aged and older individuals under scrutiny, spanning from early life stages to later adulthood compared to surveys conducted in other years. This survey endeavor contributes significantly to the holistic examination of factors influencing health during the middle-aged and older years, along with the pathways through which these influences operate, from a life course perspective.

### Multidimensional poverty assessment indicators and the computation of multidimensional poverty index

#### Assessment criteria

Table 1 lists the dimensions, specific indicators and deprivation conditions of measuring multidimensional poverty in childhood. The selection process for the five dimensions in this study was as follows: First, reference was made to the Global Multidimensional Poverty Index, jointly developed by the Oxford Poverty and Human Development Initiative (OPHI) and the United Nations Development Programme (UNDP). This index included three major dimensions: health (which comprised indicators for nutrition and child mortality), education (which included indicators for years of schooling and enrollment rates), and living standards (which encompassed six indicators: cooking fuel, sanitation, drinking water, electricity, housing, and assets) (13). Based on this framework, this study retained the health dimension. Housing conditions served as an important indicator of individuals' material living standards during childhood. Given the temporal context of the study sample (mostly born between the 1930s and 1970s), there was a significant urban-rural disparity in housing conditions during that period. Housing quality not only reflected the family's economic capacity but also profoundly influenced individuals' developmental environments and health outcomes. Additionally, given the high proportion of individuals who had experienced hunger during this period, the study elevated both nutritional status and housing conditions to independent dimensions, in order to provide a more comprehensive depiction of multidimensional poverty in childhood (14, 15). Household income directly determines economic capacity and resource allocation in China's rapid socioeconomic transition, critically shaping children's access to education and healthcare. This justifies its inclusion in multidimensional poverty measurement. Therefore, income was included as one of the dimensions for measuring multidimensional poverty in childhood (16). As an indicator of children's health, vaccination can not only effectively prevent a variety of serious diseases, reduce morbidity and mortality, but also promote children's long-term health, cognitive development and educational performance. At the same time, the difference in vaccination rates reflects the fairness of medical resource allocation and brings significant economic and social benefits (17). The selection of “vaccination” as an indicator can reflect the unequal distribution of medical resources. During periods of social unrest, the long-term bedridden or hospitalized situation of children due to health problems reflects the ability of families to cope with health risks (18). Most of the subjects in this article were born in the 1920s and 1970s. During this period, the interaction between neighbors mainly revolved around resource allocation, such as food rationing, which may have affected the quality of neighborhood relations. Individuals may be treated differently in school due to their family background during childhood, and this experience may have an impact on their social adaptability.

**Table 1:** Basic characteristics.

**Variable**	**Option**	**Number of people/average**	**Proportion (%)/standard deviation**
Before the age of 15 (including age 15), have you received any vaccines?	1 Indicates not vaccinated	1,247	10.40
	0 Indicates vaccinated	10,744	89.60
Did you spend one month or longer bedridden due to health reasons before the age of 15 (including the age of 15)?	1 Indicates bedridden due to illness	623	5.20
	0 Indicates not bedridden due to illness	11,368	94.80
Before the age of 15 (including 15), did you ever stay in the hospital for one month or longer due to health reasons?	1 Indicates hospitalized due to illness	290	2.42
	0 Indicates not hospitalized due to illness	11,701	97.58
Was the first type of residence a thatched house?	1 indicates a house made of thatch	4,263	35.60
	0 indicates the house is not made of thatch	7,711	64.40
Before you were 17 years old, how would you describe your family's economic situation relative to the average family in your community/village at that time?	1 indicates self-assessed poor economic status	4,337	36.17
	0 indicates self-assessed not poor economic status	7,654	63.83
Before you were 17 years old, was there ever a period of time when your family couldn't have enough food to eat?	1 indicates inability to have a full meal	7,813	65.16
	0 indicates ability to have a full meal	4,178	34.84
Did the neighbors get along well where you lived at that time?	1 for discord	375	3.13
	0 for harmony	11,616	96.87
Did you get bullied by the neighbor's kids when you were a child?	1 means being bullied	1,620	13.51
	0 means being not bullied	10,371	86.49
Did you get bullied by classmates when you were a child?	1 means being bullied	1,101	9.18
	0 means being not bullied	10,890	90.82
Did you often have a group of friends?	1 means yes	1,906	15.90
	0 means no	10,085	84.10
Gender	Male	6,426	53.59
	Female	5,565	46.41
Registered residence at birth	Town	1,238	10.32
	Rural area	10,753	89.68
Education level during adulthood	Primary school and below	6,855	57.17
	middle school	4,872	40.63
	Junior college and above	264	2.20
Marital status in the middle-aged and older adult	Married	10,687	89.13
	Not married	1,304	10.87
Number of chronic diseases in middle-aged and older adult	Continuous variable	0.694	1.013
Bedridden status due to illness in adulthood	Yes	1,798	14.99
	No	10,193	85.01
Age	Continuous variable	64.50	9.34

**Figure 1:**
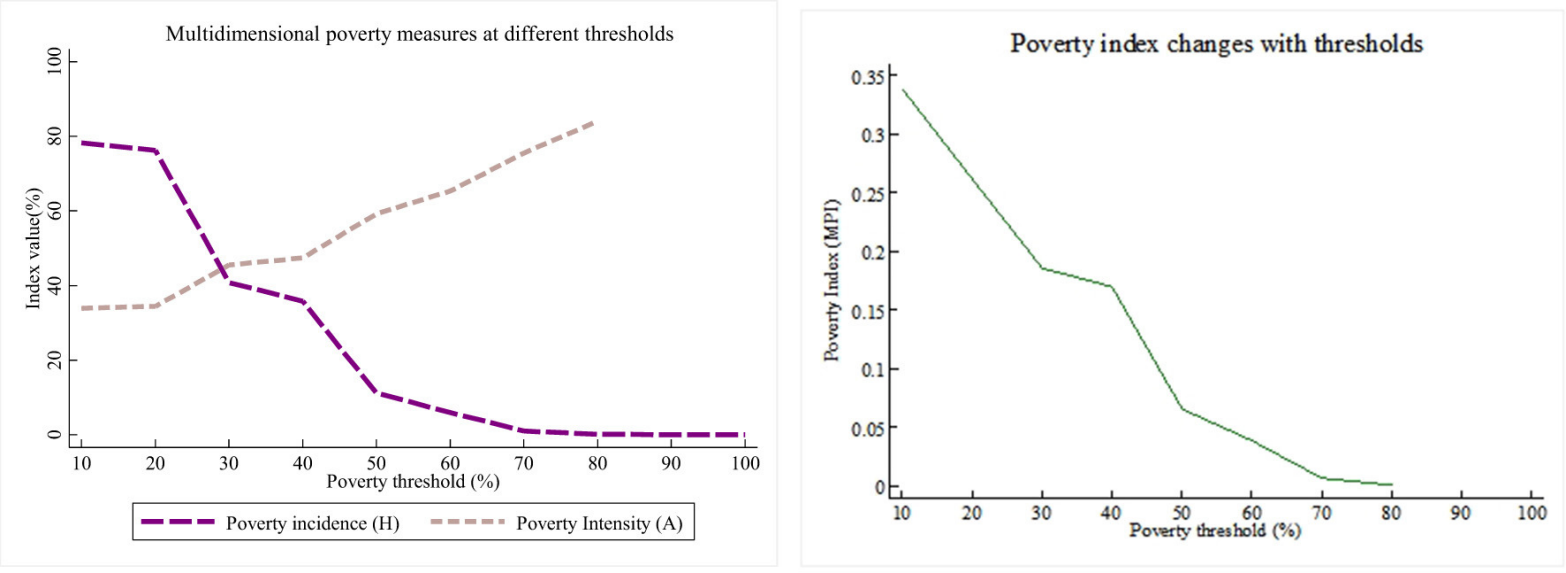
Determination of multidimensional poverty threshold.

Based on previous research (19, 20), and drawing on the changing trends of multidimensional poverty indicators at different thresholds, the selection of critical values was conducted. As shown in Figure 1, at a threshold of *k* = 30%, a good balance between poverty incidence and poverty intensity is achieved, which effectively identifies impoverished individuals and reflects the severity of poverty. Furthermore, the 30% threshold aligns with international poverty measurement standards as well as China's poverty line, providing strong theoretical and policy-based justification for its applicability. Therefore, this study used *k* = 30% as the critical value of multidimensional poverty in childhood. This study employed sensitivity analysis to determine the weights. Initially, the equal-weight method from the 2010 United Nations Human Development Report was used to assign values to each dimension (21). To ensure the robustness of the impact of weight distribution on multidimensional poverty measurement results, the weights were adjusted. The results indicated that, despite the weight adjustments, the changes in the multidimensional poverty index were minimal. Therefore, based on operational simplicity, the equal-weight method was applied to assign values to each dimension.

#### The calculation of the multidimensional poverty index (MPI) for children

Building upon the existing body of research on multidimensional poverty in China and utilizing data from the China Health and Retirement Longitudinal Study (CHARLS), this study employed the Alkire-Foster (A-F) method to measure the Multidimensional Poverty Index (*MPI*) for children in China. At that time, the A-F method is widely recognized in the field of multidimensional poverty measurement for its comprehensive information acquisition and simple operation. It is increasingly being used in research on measuring child poverty. The fundamental steps of the A-F method were as follows: Firstly, poverty identification, which included both intradimensional and interdimensional poverty identification. The former entailed identifying individuals who fell below the poverty threshold across specific dimensions, while the latter determined the number of dimensions that were considered indicators of poverty. Secondly, poverty aggregation involved summing the number of dimensions in poverty and assessing whether it qualified as multidimensional poverty. Lastly, poverty decomposition analyzed the contributions of different dimensions and indicators to the Multidimensional Poverty Index. Based on the principles and steps related to multidimensional poverty, this study calculated the incidence rate (*H*), average deprivation share (*A*), and Multidimensional Poverty Index (*MPI*) for the child population.


(1) $$\begin{array}{l} A=\frac{1}{p\times d}\sum_{i=1}^{\text{p}}ci\left(k\right) \end{array}$$

In the context of this study, *C*_*i*_(k) represents the number of dimensions by which individual, “i”is deprived when the poverty threshold is set at k, d represents the total number of dimensions.


(2) $$\begin{array}{l} \begin{array}{c} MPI=H\times A,H=\frac{\text{p}}{n} \end{array} \end{array}$$

In this context, “*p*” denotes the count of individuals experiencing multidimensional poverty, while “*n*” represents the total population under study.

### Selection of health indicators for the older adult

This study selected the ability to take care of oneself as a variable to measure the health status of older adult. The CHARLS data measures the ability to take care of oneself in the older adult through the ADL scale, which includes six indicators: “eating”, “dressing”, “going to the toilet”, “controlling urination and defecation”, “bathing”, and “getting in and out of bed”. If any of these indicators shows “needing help”, the older adult are considered “disabled”.

### Statistical methods

This study first used the probit model to analyze the long-term impact of multidimensional childhood poverty on the health status of middle-aged and older adult, and constructed a measurement model by controlling covariates such as education level and marital status. Considering the estimation bias that may be caused by sample self-selection, the propensity score matching method (PSM) was further used for robustness testing. Given that disability status has the characteristics of clear clinical diagnosis and small measurement error, this study uses it as a core health indicator to systematically evaluate the continued impact of adverse experiences in the early life course on the health of middle-aged and older adult.

## Results

### Descriptive statistics

After the removal of samples with individuals under the age of 45 and those containing missing values in key variables, a total of 11,991 valid samples remained in this study. Among the entire sample set, individuals with an agricultural household registration status at birth accounted for 89.68%, while those with an urban household registration comprised <10%.

### Measurement and analysis of multidimensional poverty in childhood

#### One-dimensional poverty measurement results in childhood

This study measured the incidence of unidimensional child poverty across five dimensions. The results showed that the main challenges facing China's child poverty focused on nutrition and income. Specifically, 65.16% (7,813) people experienced deprivation in the nutritional status dimension, while 36.17% (4,337) people experienced deprivation in the income dimension. In addition, the sample analyzed in this study showed significant levels of social participation deprivation, with a poverty rate of 31.13% (3,733). Health poverty and housing poverty rates were 16.6% and 16% respectively.

#### Results of multidimensional poverty calculation

The study focused on measuring and analyzing multidimensional child poverty using indicators such as the multidimensional poverty incidence intensity (*A*) and multidimensional poverty index (*MPI*). The analysis, based on different critical value ranges, particularly highlighted the impact of changes in the critical value (*k*) on multidimensional poverty. The result showed a significant shift in the *MPI* when *k* ≦ 30%, while changes were minimal when *k* > 30%. As *k* values increased, there was a gradual decrease in multidimensional poverty incidence, a rise in the average deprivation rate index (*A*) of multidimensional poverty, and a subsequent decrease in the multidimensional poverty index (approaching 0). The study emphasized that a higher k value led to a notable reduction in childhood multidimensional poverty, primarily due to the continuous decrease in poverty incidence.

Specifically, the study defined *k*=30% as the threshold for identifying childhood multidimensional poverty, where individuals experiencing deprivation in at least one-third of the indicators were considered in a state of multidimensional poverty. The findings revealed a childhood multidimensional poverty proportion of 40.80%, poverty intensity of 45.50%, and a multidimensional poverty index of 0.186 (Table 2).

**Table 2:** Calculation results of multidimensional poverty in childhood.

**k**	***H* incidence rate of multidimensional poverty (%)**	***A* intensity of multidimensional poverty (%)**	***MPI* multidimen-sional poverty index**
10	78.20	33.90	0.339
20	76.20	34.40	0.262
30	40.80	45.50	0.186
40	35.80	47.40	0.170
50	11.20	59.2	0.066
60	6.00	65.30	0.039
70	1.00	75.5	0.007
80	0.20	84.0	0.001
90	0		
100	0		

#### Analysis of the contribution of various indicators of multidimensional poverty in childhood

This study analyzed various indicators of multidimensional poverty among children. The results showed that, among the five dimensions, the nutritional status contributed the most to the childhood multidimensional poverty index, accounting for 41.10%, making it the primary cause of childhood multidimensional poverty. This was followed by the income dimension, which contributed 33.1%. This shows that in the early stages of life, good economic conditions and sufficient material resources are crucial to ensuring the healthy growth and dignified life of children. It is worth noting that more than three-fifths of the individuals in the sample of this study failed to meet basic living needs in childhood, and more than one-third of the individuals came from families with relatively weak economic conditions. This indicated that the generations born in China from the 1930s to the 1970s, when the country was economically underdeveloped, faced significant shortages of material resources (Table 3).

**Table 3:** Decomposition of dimensions and indicators of multidimensional poverty in childhood.

**Dimensions**	**Indicator**	**Indicator weight**	**Dimension weight**	**Indicator contribution (%)**	**Dimension contribution (%)**
Health dimension	Vaccination	0.07	0.2	2.10	4.1
	Bedridden due to illness	0.07		0.60	
	Hospitalized due to illness	0.07		1.40	
Housing dimension	Housing quality	0.2	0.2	14.10	14.1
Income dimension	Self-evaluation of economic situation	0.2	0.2	33.10	33.10
Nutritional status dimension	Experience of starvation	0.2	0.2	41.10	41.10
Social interaction dimension	Neighborhood relations	0.05	0.2	0.7	7.7
	Partnership	0.05		2.60	
	Classmate relationship	0.05		1.80	
	Opportunities for peer interaction	0.05		2.60	

### The impact of multidimensional poverty in childhood on the disability of middle-aged and older adult

This study, anchored in life course theory, bolstered regression outcomes by encompassing childhood, adulthood, and middle-to-old-age experiences. Control variables included gender, age, household registration type at birth, and educational attainment in adulthood. The findings indicated a notable 4.3% higher likelihood of developing disabilities in middle and old age for individuals who experienced multidimensional poverty in childhood compared to those who did not. Additionally, regression consistently showed higher disability likelihood among females, increasing disability with age, more pronounced disability in rural-born older adult, and decreased disability likelihood with higher adult education. Furthermore, an increase in the number of chronic diseases during middle and old age was correlated with a higher risk of disability. Notably, being married was associated with a lower likelihood of disability within this demographic (Table 4).

**Table 4:** The impact of multidimensional poverty in childhood on the disability status of middle-aged and older adult.

**Variable**	**Regression coefficients**	**Standard error**	**Marginal effect**	**95% confidence interval**
Multidimensional poverty	0.178^a^	0.031	0.043	0.117~0.238
Age	0.038^a^	0.002	0.009	0.035~0.041
Gender	−0.298^a^	0.029	−0.081	−0.260~-0.147
Bedridden due to illness in adulthood	0.401^a^	0.036	0.098	0.331~0.471
Number of chronic diseases in middle-aged and older adult	0.195^a^	0.013	0.047	0.170~0.220
Adult education level	−0.203^a^	0.029	−0.049	−0.090~-0.055
Marital status in middle-aged and older adult	−0.111^a^	0.043	−0.027	−0.195~-0.028
Place of residence at birth	−0.203^a^	0.050	−0.052	−0.301~-0.104
Adult education level	−0.203^a^	0.029	−0.049	−0.090~-0.055

“^a^” means P < 0. 001, the difference was statistically significant; Log likelihood = −5,239.9265 Pseudo R^2^ = 0.1234.

### Heterogeneity test

To analyze the heterogeneity of the impact of childhood multidimensional poverty on the disability status of middle-aged and older adult, this study grouped the research subjects by gender and household registration at birth. It then examined how these demographic characteristics moderated the effects of childhood poverty on disability outcomes later in life. The regression results were shown in Tables 5, 6.

**Table 5:** The impact of multidimensional poverty in childhood on the disability of middle-aged and older adult in rural areas.

**Variable**	**Model 1**			**Model 2**		
	**Rural areas**			**Urban areas**		
	**Regression coefficients**	**Standard error**	**Marginal effect**	**Regression coefficients**	**Standard error**	**Marginal effect**
Multidimensional poverty	0.176^a^	0.032	0.044	0.200	0.120	0.042
Age	0.039^a^	0.002	0.010	0.033^a^	0.005	0.007
Gender	−0.317^a^	0.030	−0.078	−0.114	0.095	−0.024
Bedridden due to illness in adulthood	0.386^a^	0.038	0.095	0.529	0.113	0.111
Number of chronic diseases in middle-aged and older adult	0.201^a^	0.014	0.050	0.155^a^	0.036	0.033
Adult education level	−0.206^a^	0.031	−0.051	−0.174^b^	0.083	−0.037
Marital status in middle-aged and older adult	0.022^c^	0.045	−0.026	−0.192	0.127	−0.040

“^a^” means P < 0. 001, the difference was statistically significant; “^b^” stands for P < 0.01, the difference is statistically significant; “^c^” means P < 0. 05, the difference is statistically significant; Model 1: Pseudo R^2^ = 0.1233; Model 2: Pseudo R^2^ = 0.1192.

**Table 6:** The impact of childhood multidimensional poverty on disability in middle-aged and older adult of different genders.

**Variable**	**Model 1**			**Model 2**		
	**Men**			**Women**		
	**Regression coefficients**	**Standard error**	**Marginal effect**	**Regression coefficients**	**Standard error**	**Marginal effect**
Multidimensional poverty	0.117^a^	0.043	0.026	0.247^a^	0.045	0.065
Age	0.038^a^	0.002	0.009	0.038^a^	0.002	0.010
Gender	−0.121a	0.073	−0.027	−0.295^a^	0.070	−0.086
Bedridden due to illness in adulthood	0.448^a^	0.048	0.101	0.341^a^	0.054	0.090
Number of chronic diseases in middle-aged and older adult	0.196^a^	0.017	0.044	0.196^a^	0.019	0.052
Adult education level	−0.197^a^	0.039	−0.044	−0.206^a^	0.044	−0.054
Marital status in middle-aged and older adult	−0.136	0.065	−0.031	−0.099	0.057	−0.026

“^a^” means P < 0. 001, the difference was statistically significant; Log likelihood = −2,614.2856; Model 1: Pseudo R^2^ = 0.1215 Model 2: Pseudo R^2^ = 0.1197.

#### Grouping by household registration of birth

The results showed that multidimensional childhood poverty had a significantly adverse impact on the health status of middle-aged and older adult with agricultural household registration (Table 5). In contrast, for those with non-agricultural household registration, the effect on health status was not statistically significant. Specifically, middle-aged and older adult born in rural areas who experienced multidimensional poverty in childhood had a 4.4% increased probability of disability. This finding highlighted from a specific perspective the adverse impact of China's urban-rural dual household registration system on the wellbeing of individuals with agricultural household registration. Individuals born in rural areas faced a variety of early disadvantages in their life trajectories due to insufficient public health services and infrastructure, as well as an unequal distribution of social resources. The marginalized resource status, compounded by adverse external environmental factors, exacerbated their vulnerability to household poverty. For individuals belonging to this particular group, overcoming poverty became increasingly challenging, thereby exerting a significant and lasting impact on their overall personal growth. This impact extended to their long-term health and wellbeing, resulting in adverse effects that persisted throughout their lives.

#### Grouping by gender

The results indicated that multidimensional childhood poverty had lasting adverse effects on the health of both middle-aged and older men and women, and these effects were statistically significant (Table 6). However, the detrimental effects on women's health were more pronounced. Women who had experienced multidimensional poverty in their childhood exhibited a 6.5% higher likelihood of developing disabilities, whereas men experienced a 2.6% increase in the probability of disability. These findings underscored the enduring cultural belief in male dominance and female subordination within our society, which resulted in women having limited access to adequate nutrition and educational opportunities during periods of economic disadvantage. These disadvantages gradually accumulated throughout an individual's lifespan, eventually leading to a disparity in health outcomes during later stages of life.

### Robustness test

The robustness of the regression results was ensured by conducting a robustness test on the impact of childhood multidimensional poverty on middle-aged and older adult health using Propensity Score Matching (PSM). Given the non-experimental nature of the CHARLS data used in this study, collected through questionnaire surveys, various confounding factors may have existed, potentially introducing systematic biases that could compromise the accuracy of the study results. The PSM method was employed to some extent to mitigate the influence of these confounding factors, thereby yielding relatively accurate and objective results.

The results indicate that individuals experiencing multidimensional poverty in childhood exhibit higher levels of disability (Table 7). Three matching methods were employed to estimate the average treatment effect on the treated (ATT) values of childhood multidimensional poverty on disability status in older adults. The differences in Average Treatment Effect on the Treated (ATT) values among the three methods were small, and the results were statistically significant, indicating the robustness of the estimated average treatment effects of childhood multidimensional poverty on disability status in middle and old age.

**Table 7:** Estimated results of average treatment effect of multidimensional poverty in childhood on disability of middle-aged and older adult.

	**Matching method**	**Multidimensional poverty group**	**Non multidimensional poverty group**	**ATT**	**Standard error**	***t* value**
All samples	K-Nearest Neighbor Matching	0.261	0.207	0.054	0.010^a^	5.62
	Radius matching	0.262	0.215	0.047	0.009^a^	5.20
	Caliper nearest-neighbor-matching	0.263	0.215	0.048	0.009^a^	5.23
Man	K-Nearest Neighbor Matching	0.223	0.189	0.034	0.012^b^	2.89
	Radius matching	0.223	0.186	0.037	0.011^a^	3.21
	Caliper nearest-neighbor-matching	0.223	0.187	0.036	0.011^a^	3.18
Woman	K-Nearest Neighbor Matching	0.312	0.243	0.069	0.015^a^	4.57
	Radius matching	0.314	0.232	0.082	0.015^a^	5.62
	Caliper nearest-neighbor-matching	0.312	0.231	0.081	0.015^a^	5.62
Town	K-Nearest Neighbor Matching	0.229	0.160	0.069	0.035^c^	1.99
	Radius matching	0.228	0.171	0.057	0.033	1.72
	Caliper nearest-neighbor-matching	0.229	0.167	0.062	0.033	1.87
Rural	K-Nearest Neighbor Matching	0.263	0.214	0.049	0.010^a^	4.97
	Radius matching	0.264	0.211	0.053	0.009^a^	5.59
	Caliper nearest-neighbor-matching	0.263	0.211	0.052	0.009^a^	5.54

“^a^” means P < 0. 001, the difference was statistically significant; “^b^” stands for P < 0.01, the difference is statistically significant; “^c^” means P < 0. 05, the difference is statistically significant.

Specifically, multidimensional poverty in childhood was found to have increased the probability of disability in middle and old age by 4.8%−5.4%. Additionally, it was observed that multidimensional poverty in childhood could have increased the probability of disability in middle-aged and older women by 6.9%−8.2%, and by 3.4%−3.7% for men. The probability of disability among the middle-aged and older population in rural areas increased by 4.9%−5.3%, and the probability of disability among the middle-aged and older population in urban areas increased by 5.7%−6.9%, but the statistical significance was low, with a *p* value close to 0.05.

This finding was essentially consistent with the probit regression results, suggesting the robustness of the regression outcomes.

## Discussion

The study utilized data from the China Health and Retirement Longitudinal Study (CHARLS) in 2014 and 2018 to create a robust framework for assessing multidimensional childhood poverty. This study constructed a multidimensional poverty index for childhood in five dimensions: health, household income, nutritional, housing, and social interaction. Our study revealed that individuals born between the 1930s and 1970s experienced a childhood multidimensional poverty incidence rate of 40.80%, with an average intensity of 45.50%, resulting in a Multidimensional Poverty Index (*MPI*) of 0.186. In comparison, cohorts born in India between the 1960s and 1980s had an *MPI* of 0.32 (22), indicating that while China's incidence rate was relatively high, the intensity of poverty was comparatively lower. Furthermore, longitudinal analysis demonstrates a significant decline in multidimensional poverty incidence among their offspring (born between 1980 and 2000) to 21.7% (10), reflecting the intergenerational poverty reduction effects following the improvement of the social security system after the reform and opening-up policy.

Our study aimed to comprehensively analyze poverty factors using decomposition analysis, emphasizing the significant influence of food sufficiency and income levels on childhood multidimensional poverty among individuals born from the 1930s to the 1970s in China, a period marked by limited economic growth. Material deprivation during this time significantly contributed to childhood multidimensional poverty, leading to enduring impacts on individuals' survival and development. Children's needs varied across historical epochs, with those born in eras of severe material scarcity facing predominant deprivation in material resources. Thus, when assessing the impact of childhood multidimensional poverty on middle-aged and older adult disability, understanding the role of material resource deprivation becomes crucial in explaining how childhood poverty affects disability in later life stages. During historical periods with relative material abundance, factors like parental supervision, school and domestic violence, nutritional status, healthcare access (23), and social interaction (24) emerged as significant hindrances to children's rights fulfillment. The impact of childhood multidimensional poverty depended on the era's resource shortages, influencing diverse negative impacts in adulthood. Hence, understanding the impact of childhood multidimensional poverty on middle-aged and older adult disability requires consideration of era-specific deprivation conditions experienced during childhood by research subjects, aiding in the proposal of targeted poverty alleviation measures.

This study revealed that childhood multidimensional poverty significantly affected individuals' health in middle and old age, particularly impacting their disability status. Material poverty during childhood emerged as a critical contributor to overall multidimensional poverty, exerting lasting effects on adult health and economic circumstances (25). Importantly, the mechanisms linking childhood poverty to later-life disability were closely tied to resource allocation disparities rooted in family economic status. Family income played a crucial role in children's development, with higher-income households better able to invest in their children's human capital (26). Conversely, low-income families faced disadvantages in health reserves, housing conditions, and educational resources, hindering future development (27). A favorable economic situation translated to a conducive living environment, allowing higher-income households to choose residences closer to quality schools and healthcare services. This choice facilitated access to superior educational resources and healthcare, contributing significantly to early-life health and educational resource accumulation (28). Collectively, these socioeconomic advantages lay a solid foundation with profound implications for long-term health trajectories in adulthood.

Childhood nutrition has garnered significant attention within academic circles. Despite the rapid economic growth in China, the issue of insufficient childhood nutrition continues to persist. This is primarily due to the fact that many parents still hold onto the traditional belief that merely providing an adequate amount of food is enough to ensure proper growth and development in children. Consequently, parents often fail to address the problem of imbalanced nutritional intake in their children, leading to insufficient nutritional support during the early stages of life. This inadequacy has the potential to impede proper growth and hinder optimal physical development, which may ultimately contribute to compromised adult health outcomes. Additionally, the likelihood of experiencing disability in middle and old age becomes increasingly prominent. Childhood is a critical and fundamental phase for promoting individual health. Addressing multidimensional poverty during childhood, improving material living conditions, and enhancing overall quality of life during this period not only contributes to the establishment of a strong developmental foundation for individuals but also results in significant long-term health benefits for their wellbeing (29).

Multidimensional childhood poverty exerts enduring adverse effects on the health of both males and females, with a more pronounced impact observed in females. In early agricultural societies of China, the concept of “male superiority and female inferiority” was deeply ingrained, and this feudal residue persists into modern times. Especially during the 1930s to 1970s, when China's overall economic development was relatively backward and food scarcity was widespread, women born in this period had lower family status during childhood. Limited food resources were often allocated to male family members, who were considered the primary labor force. As a result, women experienced poorer nutritional status, more difficult access to health reserves, and limited educational opportunities. This disadvantaged position in terms of health and education, compounded over time, resulted in a diffusion effect later in life, exacerbating inequalities in their life course (30). This disparity in childhood experiences led to pronounced health and resource disparities between genders. Despite better childhood nutrition and educational opportunities for males, they still faced resource scarcity, leading to adverse effects of multidimensional childhood poverty on both males' and females' health status. According to the United Nations Sustainable Development Goals, “No Poverty” (SDG 1) aims to eradicate poverty, while “Good Health and Wellbeing” (SDG 3) focuses on ensuring universal health and wellbeing, with a particular emphasis on achieving health equality across gender groups (31). This study revealed that poverty is not merely an economic issue; the multidimensional poverty experienced during childhood has a significant impact on individuals' health and wellbeing in adulthood and even in old age. This effect was particularly pronounced among women. To achieve SDG 1 and SDG 3, policymakers must focus on the health and education status of impoverished children, implementing effective interventions to ensure that poor children, particularly girls, have equitable access to resources and growth opportunities. This approach will not only contribute to the fundamental elimination of poverty but also lay a solid foundation for promoting healthy aging and gender equality. In the strategic framework of healthy aging, it is essential not only to ensure that middle-aged and older women and men have equal rights but also to prioritize middle-aged and older women as a key target of the “active aging” strategy. By doing so, we can proactively prevent the deterioration of age-related disabilities and work to reduce gender disparities in health, thereby improving the quality of life for middle-aged and older women and promoting health equity among the middle-aged and older population.

The multidimensional nature of childhood poverty had a significant impact on the disability status of middle-aged and older individuals born in rural areas. In contrast, no statistically significant effect was observed among their urban-born counterparts. This observation shed light on the systemic impact of China's dual household registration system on the rural agricultural population. Possessing an agricultural hukou at birth may have implied limited access to quality public services. Coupled with the experience of childhood poverty within a challenging external environment, it could have had long-lasting negative effects on their overall health and wellbeing. Furthermore, since the initiation of economic reforms and opening-up policies, there was a significant influx of young labor force migrating to urban areas. This trend had worsened the aging of the rural population, especially with an increasing number of older individuals being left behind in rural areas. Consequently, rural regions have exhibited poorer health indicators compared to their urban older adult counterparts, and the adverse effects of multidimensional childhood poverty have been further amplified by these demographic shifts.

## Conclusion

Based on the aforementioned research findings, the formulation of poverty alleviation policies in China should be grounded in ensuring material resources for children during their formative years. Simultaneously, a life course perspective should be adopted to contemplate the issues to be addressed and poverty alleviation policies to be crafted. Attention should also be directed toward continuous investments in adult health during their productive years and providing economic assistance during their older years. This approach not only safeguards individuals' health capital throughout their entire life span but also plays a crucial role in poverty reduction and preventing inter-generational poverty transmission within the broader society.

Rural populations and women constitute more vulnerable groups within the multidimensionally impoverished populace. China should increase its support for these economically vulnerable demographics. This could begin with efforts to promote the integration of urban and rural development, as well as the equalization of basic public services. This would involve rationalizing the distribution of financial support, educational resources, and healthcare services to rural areas, thereby eliminating the long-standing barriers in resource allocation between urban and rural regions. Furthermore, there should be an increased emphasis on promoting “gender equality” through advocacy and education, with the aim of reducing, or even eliminating, gender-based discrimination. This would promote gender equality and motivate girls from underprivileged areas and families to pursue education, thus improving the living conditions of female children.

## Limitations and shortcomings

This study has some limitations. Firstly, although we used PSM to test the regression results and address some endogeneity issues, the presence of omitted variable bias remains due to the lack of suitable instrumental variables. Secondly, due to data constraints, our selection of indicators for measuring multidimensional poverty during childhood may not be comprehensive, potentially resulting in limitations in measuring multidimensional poverty.

## Data Availability

The datasets presented in this study can be found in online repositories. The names of the repository/repositories and accession number(s) can be found below: https://charls.pku.edu.cn/.
